# Supplementary material for: Islamic and capitalist economies: Comparison using econophysics models of wealth exchange and redistribution
Source: PLoS One. 2022 Sep 22;17(9):e0275113. doi: 10.1371/journal.pone.0275113 (PMC9499301; doi:10.1371/journal.pone.0275113)
Supplement: Attachment — Submitted filename: LaTeX1.pdf [file pone.0275113.s001.pdf]

Referee report on "Islamic and capitalist economies: Comparison using econophysics models of wealth exchange and redistribution".

The manuscript contains an analysis of two types of economic systems - Islamic and capitalist, viewed through the lens of kinetic exchange models (KEM henceforth) of markets. The authors show that the repeated exchange mechanism from the KEM is useful to delineate emergence of different distributional features and they shed light on how inequality evolves.

I found the idea interesting and worth pursuing. Here are my remarks.

1. It is known point of debate in the context of KEM that generally all of such models are conservative in nature - in the sense that, the total amount of money $\sum_i m_i(t)$ remains constant for all t. The current approach I think also sheds light on how one can think about non-conservative models. I advise the author to explore this point further.

2. Relatedly, it is not entirely clear to me how the author is ensuring that the system does not explode when the model is non-conservative (each round of trading may add up to more or less wealth than before the trading occurs).

3. The model given by equation 4. has been explored in *Effects of the turnover rate on the size distribution of firms: An application of the kinetic exchange models* (Chakrabarti, Physica A, 391(23) 1, 2012, 6039-50) although in the context of applying KEM in explaining firm size distributions. A parallel can be drawn here.

4. I am curious about the distinction the author has made between Islamist economies and capitalist economies. While the nature of transaction may differ, does the eventual distribution of wealth/income also differ across them? If you have some data on income/wealth in some Islamic country and some capitalist country, it would be useful to compare their empirical distributions to motivate the idea.

   I suspect that the distributions will not differ systematically across countries. If so, the author can explore why the distributions do not differ although the nature of transactions differ.
